# Memory-less response and violation of the fluctuation-dissipation theorem in colloids suspended in an active bath

**DOI:** 10.1038/s41598-017-17900-2

**Published:** 2017-12-14

**Authors:** Claudio Maggi, Matteo Paoluzzi, Luca Angelani, Roberto Di Leonardo

**Affiliations:** 1 NANOTEC-CNR, Institute of Nanotechnology, Soft and Living Matter Laboratory, Piazzale A. Moro 2, I-00185 Roma, Italy; 2 Department of Physics and Syracuse Soft Matter Program, Syracuse University, Syracuse, NY 13244 USA; 3 ISC-CNR, Institute for Complex Systems, Piazzale A. Moro 2, I-00185 Roma, Italy; 4 Dipartimento di Fisica, Università di Roma “Sapienza”, I-00185 Roma, Italy

## Abstract

We investigate experimentally and numerically the stochastic dynamics and the time-dependent response of colloids subject to a small external perturbation in a dense bath of motile *E*. *coli* bacteria. The external field is a magnetic field acting on a superparamagnetic microbead suspended in an active medium. The measured linear response reveals an instantaneous friction kernel despite the complexity of the bacterial bath. By comparing the mean squared displacement and the response function we detect a clear violation of the fluctuation dissipation theorem.

## Introduction

The dynamics and statistical mechanics of self-propelled particles is attracting a considerable attention both from the fundamental point of view and for its potential applications^1–4^. These active particles are thermodynamically out of equilibrium as they constantly consume fuel dissipating energy in the surrounding fluid. From a dynamical point of view, however, the trajectories of non-interacting active particles show no sign of non-equilibrium as time-reversal symmetry is preserved and no entropy is produced^5^. Differently, strong deviations from equilibrium can be observed when particle trajectories result from the combination of self-propulsion and interaction forces due to other particles or external fields. Under the assumption that the external forces do not alter the “internal” propulsion mechanism, schematic models of active particles simply assume that the particle velocity is instantaneously determined by the superposition of the external forces and the “propulsion force”^4,6^. In this framework the reaction of the active particle’s velocity to the external field is instantaneous as dictated by low-Reynolds number hydrodynamics of Newtonian fluids. Differently the random propulsion force relaxes on finite time-scale which depends on the specific propulsion mechanism, for example the propulsion velocity in swimming wild-type *E*. *coli* changes abruptly during “tumbles”^7^, while in chemically propelled Janus particles the swimming direction changes gradually because of rotational diffusion^8^. This combination of an instantaneous response and “colored” noise leads, by construction, to a non-equilibrium dynamics which violates the second Kubo fluctuation dissipation theorem^9^ (FDT) already at the level of individual active particles. This can be seen as the origin of many non-equilibrium phenomena observed in active particles such as the strong deviations from the Boltzmann distribution^4,6,10,11^, the emergence of novel non-equilibrium phase transitions^12^, and the striking rectification effects induced by asymmetric boundaries and objects. For example it has been shown that swimming bacteria and Janus particles can autonomously assemble with asymmetric microstructures and form self-propelled micromachines^13–17^.

Interestingly also passive colloids, interacting with active particles, inherit the non-equilibrium properties of the active bath^18–24^ and show peculiar off-equilibrium phenomena. In fact it has been shown that these “activated colloids” violate the equilibrium equipartition theorem^20^ and are subject to an effective attraction even in presence of purely repulsive forces, deviating strongly from the Boltzmann law^21^. Moreover an active bath can induce a directed transport of colloids over micro-fabricated asymmetric barriers produced by laser litography^22,23^.

All this fundamental and applied research motivates the effort in modeling accurately the dynamics of passive colloids suspended in active baths^14,18,20,25,26^. The dynamics of these tracers is determined by the dynamics of the active particles and their interactions (steric, hydrodynamic, etc.) with the passive colloids. Adopting a simplifying approach *á la Langevin* we focus only on the degrees of freedom of the colloid and treat the active bath as a source of “noise”. In addition to this the colloid is also subject to the interactions with the surrounding fluid that introduces thermal fluctuations. We thus can model the dynamics of the colloid in the active bath with the generalized Langevin equation (GLE):

1 $${\int }_{-\infty }^{t}dt^{\prime} \,\Gamma (t-t^{\prime} )\,\dot{{\bf{r}}}(t^{\prime} )={\bf{f}}+{\boldsymbol{\eta }}+{\boldsymbol{\xi }}$$

where Γ is the friction memory kernel, **r**(*t*) = (*x*(*t*), *y*(*t*), *z*(*t*)) is the position of the colloidal particle in 3-dimensions, ***ξ*** is the “active noise” term, ***η*** is the standard Langevin thermal noise and **f** is the external force. Since the colloid is coupled to the non-equilibrium active bath we expect that the FDT is violated resulting in a Γ(*t* − *t*′) having characteristic time-scale which is different from the one of the correlation of the noise: 〈***ξ***(*t*′)***ξ***(*t*)〉. Previous experimental and numerical result^20^ suggest that ***ξ*** can be modeled by an exponentially correlated (colored) noise: 〈*ξ*_*α*_(*t*)*ξ*_*β*_(*t*′)〉 = *γ* ^2^ *D*_*A*_ *δ*_*αβ*_ *e* ^−|*t*−*t*′|/*τ*^/*τ* where *α* and *β* represent the individual Cartesian components, *γ* is the drag coefficient of the colloid, *D*_*A*_ is the “active” diffusivity, and *τ* is the relaxation time of the noise. Differently the thermal noise is assumed to be delta-correlated with 〈*η*_*α*_(*t*)*η*_*β*_(*t*′)〉 = 2*γ* ^2^ *D*_*T*_ *δ*_*αβ*_ *δ*(*t* − *t*′) where *D*_*T*_ is thermal diffusion coefficient: *D*_*T*_ = *k*_*B*_ *T*/*γ* (*T* being the temperature).

It is clear that to fully characterize the dynamics described by the GLE (1) it is necessary to design experiments aimed at measuring directly the friction kernel Γ. By combining optical tweezers-based passive and active microrheology it was shown that a bacterial bath of swimming *E*. *coli* displays a viscosity without any significant frequency dependence^19^. This indicates that the friction kernel is instantaneous: Γ = *γδ*_*αβ*_ *δ*(*t* − *t*′) so that Eq. (1) reduces to:

2 $$\gamma \,\dot{{\bf{r}}}={\boldsymbol{\xi }}+{\boldsymbol{\eta }}+{\bf{f}}$$

However this study was limited to very low bacterial densities (about 3 × 10^−3^ volume fraction) and it is not clear to what extent the optical traps can affect the dynamics of swimming bacteria.

In this work we experimentally measure the time-dependent displacement of a superparamegnetic bead in a dense bacterial bath (10^10^ cells/ml corresponding to a volume fraction of about 2 × 10^−2^). The external force is applied by using a controlled magnetic field which acts uniquely on the colloidal particle. The linear response reveals an instantaneous friction kernel validating the model of Eq. (2). Differently the mean squared displacement of the particle shows the typical combination of ballistic and diffusive behavior. This results in clear violation of the FDT which has been investigated intensively in active matter both theoretically^5,27–30^ and numerically^31,32^. We also show that the same qualitative results are obtained by numerical simulations of “run and tumble” bacteria interacting with a passive colloid. Finally our numerical results suggest that even by further increasing the bacterial density the friction memory kernel remains instantaneous while the effective drag *γ* increases substantially.

## Results

### Experiment

**Figure 1:**
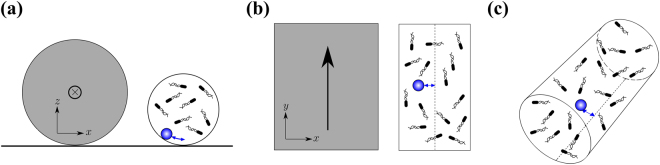
Scheme of the experimental set-up (**a**) Side view, a current-carrying copper wire (gray) is placed parallel to a glass tube containing superparamagnetic beads and bacteria. (**b**) Top view, the arrow indicates the current flow. (**c**) 3-dimensional view, the paramagnetic bead can be pulled and released by the magnetic field produced by the current.

We prepare motile *E*. *coli* cells following the protocol described in ref.^20^ (see also Methods). We use superparamagnetic microbeads of radius *a* = 3.4 *μ*m (COMPEL, Bangs). These are first diluted in deionized water and then mixed with bacteria directly on a glass slide. The final bacteria density is estimated to be ~ 10^10^ cells/ml. The bacteria-colloids solution is loaded in a microcapillary glass tube (Vitrocom) of internal radius *R* = 25 *μ*m by capillarity. The sample is left open for few minutes and then sealed with index matching oil. The filled capillary tube is then placed parallel to a copper wire (radius 200 *μ*m) at an approximate distance of 100 *μ*m (see Fig. 1). The copper wire is connected to a computer-controlled current generator. This set-up allows the combined measurement of the response of the microbeads subjected to a controlled magnetic field and the measurement of its spontaneous active dynamics in absence of external perturbations.

#### Fluctuations

**Figure 2:**
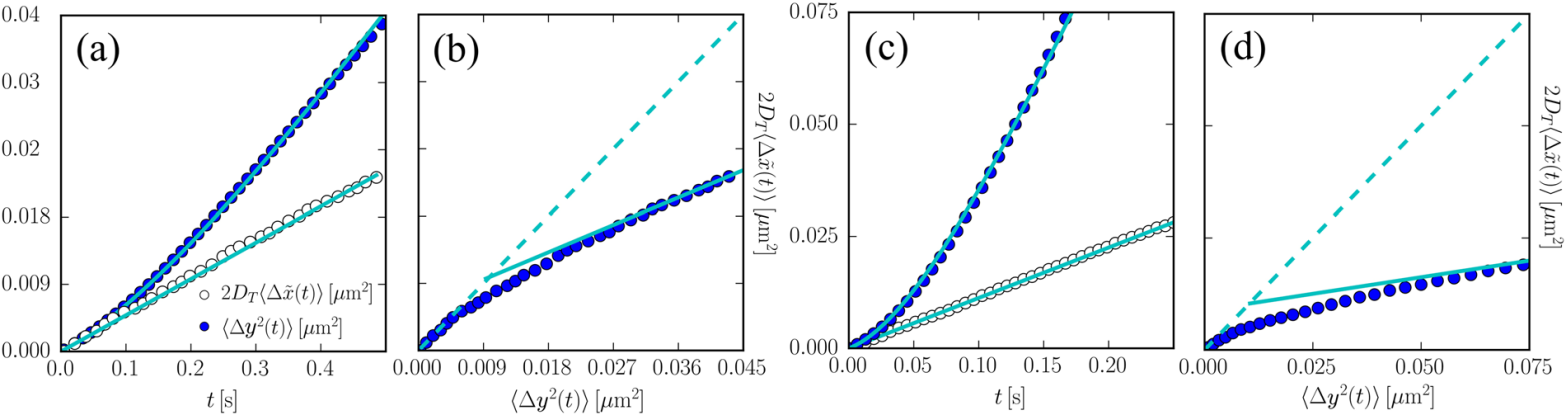
(Left panels-Experimental data) (**a**) Mean squared displacement (full symbols) and normalized response (open symbols) as a function of time for a superparamagnetic bead surrounded by swimming bacteria. (**b**) FD-plot of the response plotted as a function of the mean-squared displacement showing a clear FDT violation. (Right panels-Simulation data) (**c**) and (**d**) Same as (a) and (b) respectively for the simulation data for a passive beads surrounded by bacteria in a 2-dimensional geometry and perturbed by a constant force in the response measurement.

**Figure 3:**
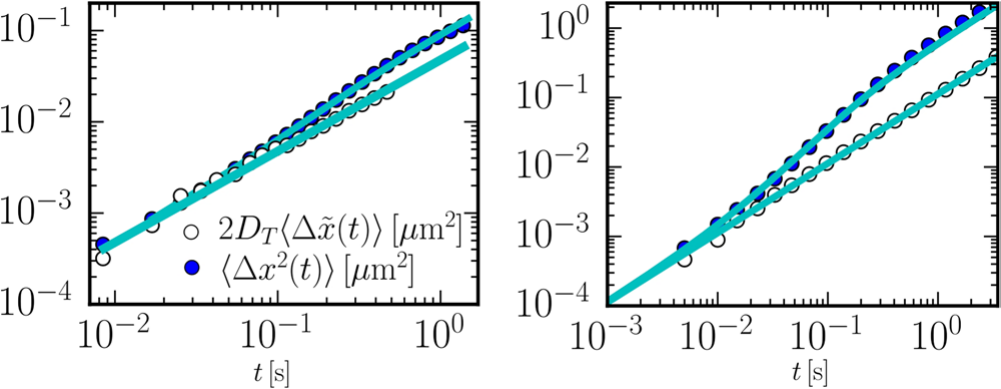
(Left) Same experimental data as in Fig. 2(a) shown in double log-scale. (Right) Same simulation data as in Fig. 2(c) shown in double log-scale.

When the current is zero no external field acts on the super paramagnetic particles. In this situation the colloids sediment at the bottom of the capillary and fluctuate because of the collisions with swimming bacteria and because of thermal agitation. We characterize the active dynamics of one single colloid one at a time in absence of external filed by collecting bright field images using a 20× microscope objective (NA = 0.25). We obtain particle trajectories by center of mass tracking directly while streaming images at a rate of 100 fps. To characterize the dynamics of the microbead in absence of external perturbation we measure the mean squared displacement (MSD) along the *y* axis 〈Δ*y* ^2^(*t*)〉 where no force is present (differently along *x* the curvature of the capillary and gravity result in an elastic force as discussed below). These measurements are obtained by averaging over about 600 trajectories (lasting for 1 s) of the same bead. The MSD for one single bead is reported in Fig. 2(a) (full symbols, see also in Fig. 3) and show the typical transition from a ballistic behaviour at short times followed by a diffusive behaviour at longer timescales (see Fig. 3) These data can be fitted very well by the theoretical MSD obtained from Eq. (2):

3 $$\langle {\rm{\Delta }}{y}^{2}(t)\rangle =2{D}_{T}t+2{D}_{A}[t-\tau \mathrm{(1}-{e}^{-t/\tau })]$$

as shown by the full line in Fig. 2(a). Fitting by Eq. (3) allows also to extract the parameters of interest, i.e. the thermal diffusivity *D*_*T*_ = (2.43 ± 0.02) × 10^−2^ *μ*m^2^/s, the active diffusivity *D*_*A*_ = (3.55 ± 0.03) × 10^−2^ *μ*m^2^/s and the relaxation time *τ* = 0.22 ± 0.03 s. The thermal diffusion constant can be used to extract the mobility of the particle as *μ* = *D*/(*k*_*B*_ *T*) = 5.92 ± 0.06 *μ*m/(s pN) which is the inverse of the drag coefficient *μ* = *γ* ^−1^. The measured *μ* is about a factor two smaller than the bulk mobility value and this is consistent with an increase of the drag caused by the presence of the capillary wall^20,33^.

**Figure 4:**
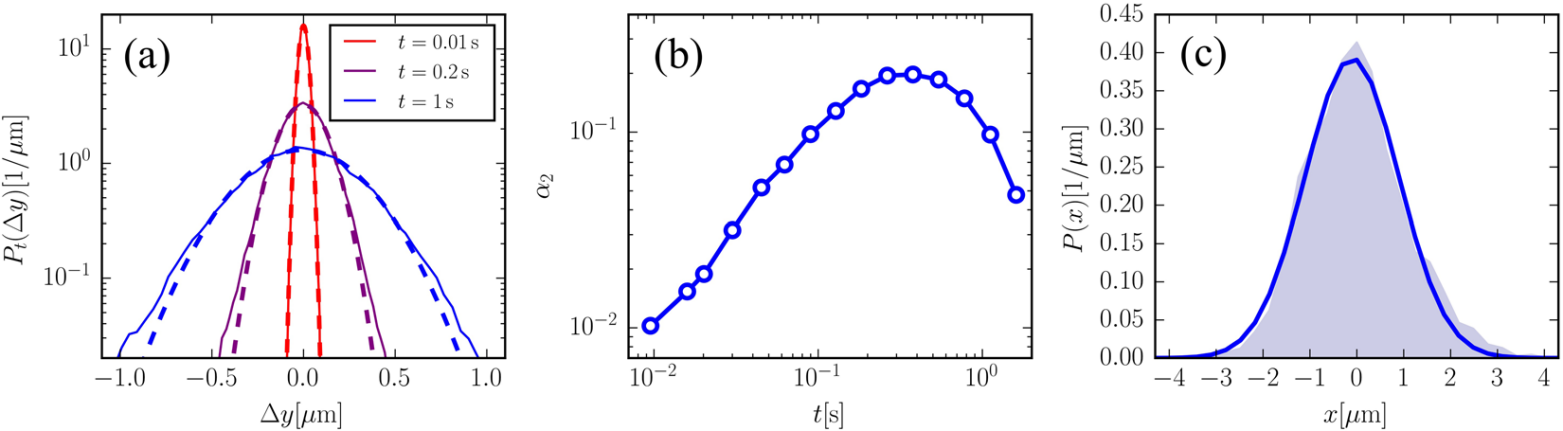
(Experimental data) (**a**) Probability distribution of the displacements along *y* (full lines) at different times (see legend). The dashed lines are fits with Gaussian functions. (**b**) Non-Gaussian parameter as a function of the time lag *t* reaching a maximum at *t* ≈ *τ*. (**c**) Probability distribution of the bead’s position along *x* (shaded area). The full line is a fit with a Gaussian.

It is also interesting to consider the statistics of displacements in absence of external forces. According to Eq. (2), (see also ref.^34^) the displacement of the particle results from the combination of the active noise ***ξ*** and the thermal noise ***η***. Since ***η*** is Gaussian distributed any deviation from the Gaussian in the displacement distribution has to be attributed to ***ξ***. In Fig. 4(a) we show the probability distribution of displacements along the *y*-axis at different time lags *t* indicated by *P*_*t*_(Δ*y*). It is evident that as *t* grows the deviation from the Gaussian fit (dashed lines in Fig.  4(a)) become more evident showing that *P*_*t*_(Δ*y*) develops “fat tails”. To quantify this effect we compute the non-Gaussian parameter as^35^: *α*_2_(*t*) = 〈Δ*y* ^4^(*t*)〉/(5〈Δ*y* ^2^(*t*)〉) − 3/5. This is shown in Fig. 4(b) and it is found to increase and then decrease as *t* grows. The parameter *α*_2_ reaches a maximum at the characteristic relaxation time of the active force *t* ≈ *τ* indicating that ***ξ*** is the responsible for the non-Gaussian behavior of *P*_*t*_(Δ*y*). Our results are in qualitative agreement with the results of ref.^36^ where robust exponential tails where found in the distribution of displacements of tracer particles suspended in an active bath of swimming algae.

Finally, for the the discussion that follows, it is also important to look at the (static) position distribution along *x*. Being in a cylindrical capillary the bead is subjected to a nearly elastic force *f*_*e*_ directed along *x* due to the combination of the confinement and gravity as discussed in ref.^20^. This force is given by by *f*_*e*_ = −*kx* where the elastic constant *k* = *mg*/(*R* − *a*) is determined by the buoyant mass of the particle *m* and the acceleration due to gravity *g*. The effect of this force is to confine the particle’s motion along *x*. This is seen in the probability distribution of the colloid’s *x*-coordinate *P*(*x*) reported in Fig. 4(c). This figure shows that *P*(*x*) is nearly Gaussian-distributed (as also found in ref.^20^) with a variance 〈*x* ^2^〉 = 1.01 ± 0.06 *μ*m^2^. Following the theory of ref.^20^ we can compute the variance from the formula 〈*x* ^2^〉 = (*D*_*T*_ + *D*_*A*_)/(*μk*) = 0.9  ±  0.2 *μ*m^2^ that is compatible with the measured value (the large uncertainty is due to the uncertainty on the particle’s density). The value of *k* could be tuned by changing the particle size and/or density or by using capillary tubes with smaller radii.

#### Response

When the electric current flows a magnetic filed is generated and this allows to measure the time-dependent response of the superparamagnetic particle. The magnetic field acts on the particle inducing a magnetic dipole moment and that interacts with the magnetic field itself. In this way the particle is attracted toward regions where the magnetic field is more intense. The force on the superparamgnetic bead subject to the magnetic field generated by a current-carrying wire takes the form^37,38^:

4 $${f}_{m}=({\mu }_{0}{a}^{3}\chi )\frac{{I}^{2}}{3\pi {r}^{3}}$$

where *I* is the current, *μ*_0_ is the magnetic permeability of vacuum, *χ* is the (dimensionless) magnetic susceptibility of the bead and *r* is the distance from the center of the wire. This force acts on the *x*-axis and pulls the particle toward the wire. By knowing the susceptibility^37^ *χ* = 0.170 ± 0.07 and the current *I* = 0.5 A we can estimate the force acting on a bead placed in the center of the capillary tube *f*_*m*_ = 0.029 ± 0.01 pN. For small displacements ≤ 0.5 *μ*m near the center of the capillary, as those observed in this experiment, this force is *f*_*e*_ ≈ 4 × 10^−3^ pN and therefore can be neglected respect to the magnetic force *f*_*m*_. However after having induced a displacement we switch off the magnetic field and this elastic force brings back the particle to the initial position making the capillary a convenient geometry for taking repeated measurement on the same bead.

**Figure 5:**
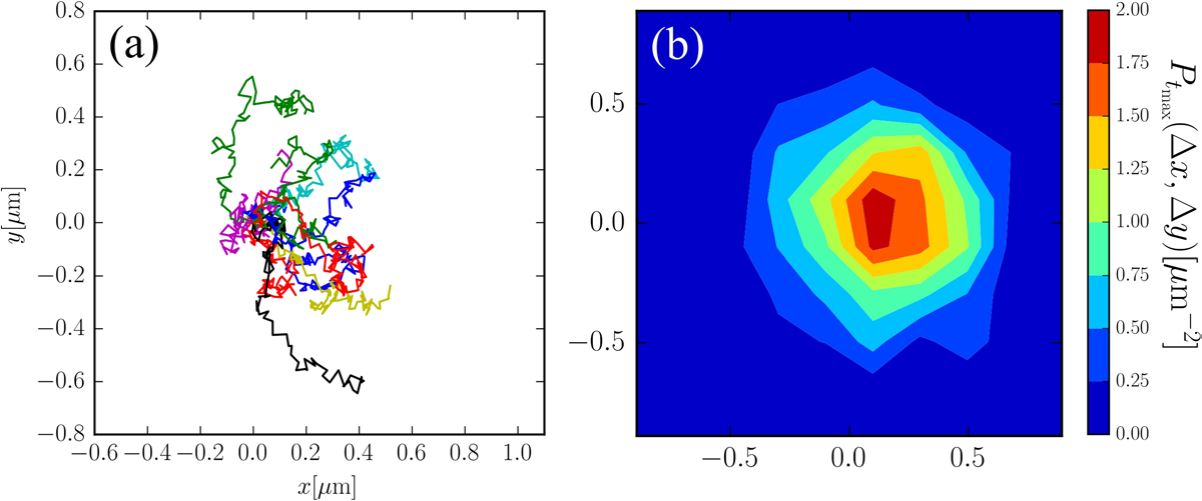
(Experimental data) (**a**) Trajectories of the same particle obtained by repeatedly switching on the field at time *t* = 0 s (all trajectories are rescaled so that the position of the particle coincides with the origin at *t* = 0). An evident drift of the particles along the magnetic field gradient (along *x*) is observed. (**b**) Probability distribution of the final displacement (*t* = 1 s) after switching the field on at *t* = 0 s. The probability peaks at Δ*x* > 0 while the peak is symmetric with respect to *y* = 0.

To perform the measurements the current is switched on at time *t* = 0 and kept constant for 1 s during which we measure the displacement of the particle along *x*. After 1 s the current is switched off and the particle is released. This procedure is repeated 600 times and the resulting displacements are averaged to obtain the average displacement 〈Δ*x*(*t*)〉 induced by the external force. Some of the trajectories obtained by the tracking of one single bead are displayed in Fig. 5(a) which shows how the external field induces a net drift of the particle from their initial position (*x* = 0) towards the right (*x* > 0). This is even more clear when we compute the probability of the displacement *P*(Δ*x*, Δ*y*) after a time *t* = 1 s (see Fig. 5(b)) which is clearly peaked around positive Δ*x* while being fairly symmetric with respect to the *y* axis.

The results obtained by averaging these measurements are shown in Fig. 2(a) (open symbols) for the same bead whose MSD is shown in the Figure. The measured 〈Δ*x*(*t*)〉 can be fitted very well with a straight line 〈Δ*x*(*t*)〉 = *vt* (constant speed drift) with *v* = 0.201 ± 0.005*μ*m/s. This confirms that the memory friction kernel of colloids in active baths is indeed instantaneous. Note that to plot 〈Δ*x*(*t*)〉 together with the MSD in Fig. 2(a) we have normalized it by the fitted speed *v* defining $\langle {\rm{\Delta }}\tilde{x}(t)\rangle =\langle {\rm{\Delta }}x(t)\rangle /v$ and we have multiplied this by the thermal diffusivity *D*_*T*_. In this way the MSD and the displacement follow each other at small-times and separate over longer timescales. Moreover having estimated the force we can extract the mobility as *μ* = *v*/*f*_*m*_ = 6.8 ± 0.5*μ*m/(s pN) which is in fairly good agreement with the measurement of *μ* obtained from the MSD given above.

Having measured the MSD and the displacement following the perturbation we can detect a clear difference between the noise kernel and the friction kernel. To show this we build the fluctuation-dissipation plot (FD-plot)^39^ in which we report the normalized response $2{D}_{T}\langle {\rm{\Delta }}\tilde{x}(t)\rangle$ versus the MSD where time is a parameter as shown in Fig. 2(b). If the system were in equilibrium the data would follow the dashed line in Fig., i.e. $2{D}_{T}\langle {\rm{\Delta }}\tilde{x}(t)\rangle =\langle {\rm{\Delta }}{x}^{2}(t)\rangle$. Differently the non-equilibrium dynamics generated by the active bath leads to a quasi-equilibrium regime at timescales *t* ≤ 0.1 shorter than *τ*, where the FDT is valid, followed by a clear violation of the theorem at longer timescales where $\mathrm{2(}{D}_{T}+{D}_{A})\langle {\rm{\Delta }}\tilde{x}(t)\rangle =\langle {\rm{\Delta }}{x}^{2}(t)\rangle$ characterized by an enhanced diffusivity. This can be translated in an effective temperature that, over long timescales, is higher than the bath temperature: *k*_*B*_ *T*_eff_ = (*D*_*T*_ + *D*_*A*_)/*μ* = (2.5 ± 0.1) × *k*_*B*_ *T* for the data in Fig. 2(b).

### Simulations

The numerical simulations are performed by considering one single spherical colloidal particle of radius *a* immersed in a bath of bacteria modeled as self-propelling dumbbells following a “run and tumble” dynamics. Both particles and bacteria move in a 2-dimensional box with periodic boundary conditions. All interactions between bacteria and between bacteria and particles are modeled by steric repulsive forces. In addition we apply a constant force to the colloidal particle when we want to measure the response function. We include Brownian motion only for particles dynamics and neglect hydrodynamic interactions^14,21^. A detailed description of the simulation can be found in refs^13,21^. We first focus on the case where the bacterial density is low (*ρ* = 1.2 × 10^−2^ *μ*m^−2^) which yelds results more similar to experimental case.

The axial MSD of the particle is shown in Fig. 2(c) (full symbols) and is qualitatively very similar to the one found in experiments. These numerical data can also be fitted very well with Eq. 3 as shown by the full line in Fig. 2(c) and in Fig. 3. The resulting fitting parameters are *D*_*A*_ = 0.260 ± 0.004μm^2^/s, *τ* = 0.071 ± 0.003 s and *D*_*T*_ = 0.056 ± 0.002 *μ*m^2^/s. We note that the fitting parameters from the simulation data are of the same order of magnitude of the experimental ones although the active diffusivity *D*_*A*_ is considerably higher in simulations than in experiments as found also in ref.^20^ Also for simulations we extract the mobility as *μ* = *D*_*T*_/*k*_*B*_ *T* = 13.8 ± 0.4 *μ*m/(s pN). As expected at such low density this is very close to the bare mobility used in the simulation *μ* = 13.6 *μ*m/(s pN). For measuring the externally induced displacement we apply a constant force *f* = 0.2 pN directed along the *x*-axis at *t* = 0. This is kept constant for 1 s, during which we measure the time dependent displacement, and the procedure is repeated for 10 times. The normalized displacement $\langle {\rm{\Delta }}\tilde{x}(t)\rangle$ is reported in Fig. 2(c) (open symbols) and reveals again a constant speed drift corresponding to an instantaneous friction kernel. By fitting the displacement with a constant-speed motion 〈Δ*x*(*t*)〉 = *vt* we get *v* = 2.68 ± 0.05 *μ*m/s from which we obtain the mobility *μ* = *v*/*f* = 13.7 ± 0.3 *μ*m/(s pN). From the combination of the numerical MSD and displacement we can build the FD-plot shown in Fig. 2(d). This shows a form that is qualitatively similar to the one found in experiments with the FDT being violated at long timescales. However *D*_*A*_ is higher in simulations than in experiments and consequently we get a higher effective temperature *k*_*B*_ *T*_eff_ = (*D*_*T*_ + *D*_*A*_)/*μ* = (5.6 ± 0.2) × *k*_*B*_ *T*.

**Figure 6:**
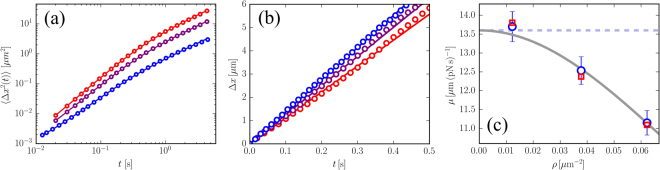
(Simulation data) (**a**) Symbols represent the MSD of a particle in a bacterial bath at three different densities (0.012, 0.038 and 0.062 *μ*m^−2^ from bottom to top), the lines are fits with Eq. (3). (**b**) Time-dependent displacement (symbols) of the particle induced by same the external field (turned on at time *t* = 0s) in numerical simulation at three different densities (same as in (a)). The full lines are a linear fits passing through zero. (**c**) Resulting mobility (circles) from the drift velocity found in (**a**) compared with the mobility from the MSD data (squares) shown in (b) (error bars not show). The mobility smoothly decreases from the bare mobility (dashed line) upon increasing density as suggested by the fitting (full line).

Upon increasing the density of the active bacteria we observe that the MSD increases considerably in amplitude (see Fig. 6(a)). These data can be very well fitted with Eq. (3) allowing to estimate the parameters *D*_*A*_, *D*_*T*_ and *τ*. We find that *τ* and *D*_*A*_ both increase respectively from 0.10 to 0.18 s and from 0.26 to 3.3 μm^2^/s upon increasing *ρ* from 0.012 to 0.062 μm^-2^. Differently *D*_*T*_ systematically decreases from 5.6 × 10^−2^ to 4.5 × 10^−2^ μm^2^/s in the same density range. This indicates that the effective mobility *μ* = *D*_*T*_/*k*_*B*_ *T* of the particle in the bacterial bath,probed by the thermal fluctuations, decreases upon increasing the density. The response measured upon changing density remains instantaneous resulting in a linear drift of the particle following the activation of the external field (see Fig. 6(b)). While this generalizes the validity of Eq. (2) to the moderately high-density regime we also observe that the effective mobility of the particle found in simulations decreases systematically confirming the trend observed in the thermal diffusivity. By fitting the data in Fig. 6(b) with straight lines we obtain the drift speed of the particles from which we can estimate again the mobility. This compares very well with the one extracted from *D*_*T*_ as shown in Fig. 6(c). These data can be fitted with a simple polynomial equation *μ* = *μ*_0_/(1 + *αρ* + *βρ* ^2^) with *μ*_0_ being the bare mobility of the particle and with *α* > 0, *β* > 0. This suggests that, at very low densities, the mobility approaches the bare mobility of the particle set by the fluid and that at high *ρ* the packing of the bacteria becomes important lowering significantly the mobility *μ*.

## Discussion

We have studied numerically and experimentally the time-dependent response and fluctuations of a particle immersed in a bacterial bath. We have shown that this response is instantaneous which, in combination with the persistent behaviour of fluctuations, leads to a dramatic breakdown of the fluctuation-dissipation theorem. Our results further confirm the validity of the model that is currently used to describe the effect of an active bath on a passive particle: the (thermal) Langevin dynamics is simply modified by adding active (persistent) forces without changing the response function. Moreover our results show some intriguing analogy with recent simulations on active red-blood cells membrane in which it has been shown that the response function of the membrane in presence of active fluctuations is the same that in complete absence of activity while the fluctuations change substantially in the two cases^40^. This is consistent with our results in which, by adding the activity of the bacterial bath, the response of the probe particle does not change. Further experimental, theoretical and numerical studies would be welcomed to firmly asses how general is the scenario in which the active bath leads to non-equilibrium fluctuations but to an equilibrium-like response.

## Methods

### Bacteria preparation

*E*. *coli* cells (MG1655) are grown overnight at 33 °C in tryptone broth (TB, Difco) containing 1% tryptone and 0.5% NaCl. The saturated culture is then diluted 1:100 (50 *μ*l in 5 ml) into fresh medium and grown at 33 °C until OD600 = 0.4 (optical density at 600 nm wavelength) is reached. This optical density corresponds to to the half of the extent of the exponential growth phase (i.e. the so-called “middle-log” phase^41^). Bacterial cells are then harvested from culture media by centrifugation at 2200 rpm for 10 minutes at room temperature. The pellet is resuspended by gently mixing in a pre-warmed motility buffer composed by 10 mM potassium phosphate, 0.1 mM Na-EDTA (pH 7.0), 76 mM NaCl and 0.002% Tween-20^42^. This motility buffer does not sustain cell replication at room temperature, so the population remains constant in the experiment that follows.
